# Harm reduction and equity of access to care for French prisoners: a review

**DOI:** 10.1186/1477-7517-5-17

**Published:** 2008-05-21

**Authors:** Laurent Michel, M Patrizia Carrieri, Alex Wodak

**Affiliations:** 1 Health and Medical Research National Institute, Research Unit 669, Paris, France; 2 University of Paris-Sud and University Paris Descartes, umr-s0669, Paris, France; 3 Emile Roux Hospital, Limeil-Brévannes, France; 4 Inserm umr912 "Economic & Social Sciences, Health Systems & Societies", Marseille, France; 5 Southeastern Health Regional Observatory (ORS-PACA), Marseille, France; 6 St. Vincent's Hospital, Sydney, Australia

## Abstract

**Background:**

Despite France being regarded as a model of efficient harm reduction policy and equity of access to care in the general community, the health of French inmates is a critical issue, as harm reduction measures are either inaccessible or only partially implemented in French prisons.

**Method:**

Using specific inclusion and exclusion criteria, information was collected and analyzed about HIV, HBV and HCV prevalence, risk practices, mortality, access to harm reduction measures and care for French prison inmates.

**Results:**

Data about the occurrence of bloodborne diseases, drug use and access to care in prisons remain limited and need urgent updating. Needle exchange programs are not yet available in French prisons and harm reduction interventions and access to OST remain limited or are heterogeneous across prisons. The continuity of care at prison entry and after release remains problematic and should be among the primary public health priorities for French prisoners.

**Conclusion:**

Preventive and harm reduction measures should be urgently introduced at least as pilot programs. The implementation of such measures, not yet available in French prisons, is not only a human right for prison inmates but can also provide important public health benefits for the general population.

## Introduction

There is increasing acknowledgement that the health of prison inmates is both a critical issue in its own right and a public health concern, as after release inmates may discontinue HIV care or opioid substitution treatments and be more inclined to engage in unsafe injecting practices. The physical and mental health of persons entering prison is often poor and may be further impaired after entry by a combination of factors including high risk sexual and drug injecting behavior [1-4], violence, non-consensual sex [5] and mental illness [6-8].

Many inmates cycle in and out of prison repeatedly, increasing the likelihood that any infections contracted in prison could soon affect the general community. Therefore, careful surveillance of infections in prison populations could help to predict future outbreaks of infections in the general population [9,10].

Moreover, in many countries in the world today, a considerable percentage of people entering prison are drug dependent prior to incarceration and many of these continue to use drugs, generally by injection, after entering prison [11-15]. The major 'currencies' used in prisons around the world are sex and drugs. It is very difficult for prison authorities around the world to ever acknowledge the fact that vigorous and expensive efforts to prevent drugs from entering prison have very limited effect and may render drug injection which does occur even more hazardous. When needle exchange programs (NEP) are unavailable in the prison setting, HIV-HCV risk behaviors may be extremely frequent as documented by some studies reporting risk behaviors [1-4] and may result in HIV – HCV seroconversions in the prison setting [11,16-19]. Injecting equipment used in prisons is excessively worn, thereby increasing the risk of blood borne viral transmission. Equipment sharing generally occurs with many partners from diverse geographical and social networks, further increasing the potential public health impact. Studies underestimate the extent of the problem as seroconversion often occurs after release although the infection occurred during the period of incarceration.

In addition, lack or difficult access to condoms also contributes to an increased risk of HIV or HBV seroconversion due to high risk sexual behaviors including sexual assault [20], while frequent movements of inmates within the prison system and the almost inevitable over-crowding of prisons facilitate the spread of tuberculosis [21-26].

Despite the increasing interest in health in prisons, the inadequate access to preventive measures and the lack of an efficient and comprehensive system of care (including care for psychiatric co-morbidities), make the need to improve correctional health services and outcomes a matter of urgency.

In 1996 France was faced with an alarming HIV epidemic among drug users. HIV prevalence among injecting drug users was estimated to be 40% [27], forcing the adoption of harm reduction including the scale up of NSPs (needle syringe programs) and the introduction of opioid substitution treatment (OST) – buprenorphine in primary care and methadone, also available in primary care after dose stabilization. Within 10 years, the benefits of this approach were self-evident: a 5-fold reduction in overdose deaths [28] and a four-fold reduction in HIV prevalence (11%) in drug users [29]. The decrease in HCV prevalence among drug users – from 70% to 60% – was less impressive [29].

Despite the World Health Organization (WHO) statement *"All prisoners have the right to receive health care, including preventive measures, equivalent to that available in the community"*[30], NSPs and easy access to condoms are not yet available in French prisons while access to and varieties of available OST vary greatly from one prison to another. The variability in prison OST is partly attributable to the specific health policy of some prisons but also reflects the difficulties of employing adequate numbers and assuring quality of staff.

Data from the French correctional system about drug use, risk behaviors of inmates, HIV and HCV seroprevalence, access to OST, antiretroviral treatment and post-exposure prophylaxis is scattered throughout many different reports or papers. Most are in French with only a few papers in English and some of these are obsolete as they pertain to the era before highly active antiretroviral treatment (HAART).

The objective of this review is to summaries the health data available regarding French prison inmates, to indicate the need for future research to improve the health status of prisoners and to encourage access to health care for the inmate population equivalent to standards available in the community.

## Materials and methods

### Criteria for considering studies for this review

Literature was reviewed starting from the most recent reports and papers available on the internet as well as those presented at French conferences dealing with HIV, HCV, harm reduction, or prevention in French prisons. Using the references cited in these papers and reports it was possible to retrieve still other studies and reports including those belonging to grey literature.

Once all the documents were accessible we used the following inclusion criteria: studies documenting HIV, HCV and HBV; suicide rates; drug use and alcohol consumption; HIV-HCV-HBV risk practices; access to HAART and opioid substitution treatment and continuity of care both during prison stay and after release; recidivism rates; knowledge, attitudes and practices towards harm reduction measures such as NEP or condom distribution.

Though more related to psychiatric co-morbidities, suicides were included in this review because of the link between drug use and suicide risk. Data collected from inmates or health care professionals working in prisons were included in these studies.

Exclusion criteria excluded studies focusing on psychiatric comorbidities and care or other conditions not directly related to bloodborne transmission.

Moreover epidemiological studies whose methodology for data collection remained undefined or inaccurate were excluded from this review. Only data pertaining to the last 15 years were included in this review.

## Results

As of January 1^st ^2006, 59,522 inmates were incarcerated in French prisons. Among them, 19,732 (33.8%) were awaiting sentence and 38,612 (66.2%) had already been sentenced, 14.4% of those for drug related offences [31].

In 2005 [32], 36,264 had been sentenced for drug related offences (23,760 prison terms including 8,334 imprisonments and 15,426 partial or total suspended sentences, with or without probation), 12,564 for possession/acquisition of drugs, 13,104 for illicit use, 1,943 for trafficking, 6,571 for trading/transport and 1,924 for offering drugs). The mean duration of imprisonment for drug related offences was 13.9 months (3.4 months for a single offence, 15.3 months for multiple offences), 11.4 months for acquisition and detention, 6.1 months for illicit drug use, 27.9 months for trafficking (import, export), 15.7 months for trading/transport, and 9.5 months for offering drugs.

Since 1994, health in French prisons has been the responsibility of the Ministry of Health. Care provision for inmates is organized in cooperation with neighborhood hospitals independently from the prison administration. Care is freely available with inmates getting full benefits from social insurance from the time they enter prison. After release, inmates (and their family) can still benefit from free health care for four years. Costs of screening, treatment and staff are included in the global budget of the hospitals. Resources allocated to care for inmates in need have been increased frequently, especially since 1994.

### Health status at prison entry

#### HIV, HCV, HBV at prison entry

At entry to a French prison, all inmates must undergo a comprehensive medical examination. The Ministry of Health collected the available medical data from these examinations in 1997 and 2003 for all prisons in France [33]. The duration of data collection in 2003 varied according to the size of the prison (2 weeks for a large prison with 9,272 new inmates in 2002, 1 month for jails with more than 600 entrants in 2002, 2 months for prisons with between 300 and 600 entrants, 3 months for jails with fewer than 300 entrants in 2002).

The proportion of inmates tested for HIV and HBV at admission (see table 1) decreased from 1997 to 2003 (46.5% in 1997 vs. 40.0% in 2003 and 25% in 1997 vs. 20.5% in 2003 respectively), but increased for HCV (19.7% in 1997, 27.4% in 2003). The proportion of inmates vaccinated against HBV at entry increased from 13.7% in 1997 to 31.3% in 2003.

**Table 1:** Access to care and HIV, HBV and HCV status at prison entry and during prison stay.

	**Mouquet**	**Mouquet**	**Remy**	**Remy**	**Drassif**
	1997 (%)	2003 (%)	2000 (%)	2003 (%)	2005 (%)
	135 prisons Incoming inmates*	134 prisons Incoming inmates*	85 prisons 27 245 inmates**	88 prisons 31215 inmates**	8 prisons Incoming inmates***
HCV positive test	4.4^a^	4.2^a^	6.3^b^	6.9^b^	5.9 ^b^
% of inmates screened	20	27	ND	ND	38
HBV positive test	2.3^a^	0.8^a^			3.4 ^b^
% of inmates screened	25	20			37
HIV positive test	1.6^a^	1.1^a^			0.6 ^b^
% of inmates screened	46	40			41
HAART at prison entry	0.8	0.5			1.4
HCV treatment (total number/1 year)			164	297	171

*study period : from 2 weeks to 3 months depending of the size of the prison

** cross-sectional study

***study period : 2005

^a^self-reported serostatus among those who reported to have had already been screened

^b ^positive test

Prevalence of HIV at entry (self-reported) decreased from 1.6% in 1997 to 1.1% in 2003, with 0.8% vs. 0.5% reporting receiving HAART respectively.

Prevalence of HCV and HBV at entry (self-reported) decreased respectively from 4.4% in 1997 to 4.2% in 2003 and 2.3% in 1997 to 0.8% in 2003.

HIV prevalence for inmates reporting a history of drug injection decreased from 9% to 5% between 1997 and 2003 [33].

In a national postal survey of prison medical services for HCV screening and care conducted in 2000 and again in 2003, authors received answers from 88 out of 172 prisons [34].

In this survey, HCV prevalence estimates remained quite stable: 6.9% in 2003 and 6.3% in 2000.

One survey [35] conducted in 2005 and including 8 prisons in Paris and its suburbs showed that among prisoners at time of incarceration who reported having had already been tested for HIV (41%) for HCV (38%) and for HBV (37%), 0.6% reported to be HIV-positive, 5.9% HCV-positive and 3.4% HBV-positive. An important heterogeneity existed among prisons concerning the rates of inmates reporting to have had already been tested (from 29% to 80% for HIV, 17% to 80% for HCV and 27% to 80% for HBV).

#### Drug and alcohol use at entry

Information on drug or alcohol use at entry is based on self reported data. No data from urine or blood drug screen were available.

At entry, 33.3% (1997) and 30.9% (2003) of inmates reported excessive alcohol use (>4 alcohol units/day for men and >2 alcohol units for women and/or > 4 consecutive alcohol units at least once a month) [33].

Sahajian et al. [36] described the population of prisoners at time of incarceration in prisons in the area of Lyon between January 1^st ^and December 31^st ^2003. Among them, 68.5% reported no regular employment in the previous 12 months and 52.8% had previously been imprisoned. More than 64.0% of inmates reported regular tobacco use, 16.5% cannabis use, 16.1% alcohol use, 25% psychotropic medication, and 4.1% reported drug use (heroine, cocaine or synthetic drugs). Moreover, 42.0% of drug users reported polydrug use or dependence on 2 or more drugs (see table 2).

**Table 2:** Substance use and access to care at prison entry.

	**Mouquet**		**Sahajian**	**Lukasiewicz**	**Oppidum**	**Feuillerat**		
	1997 (%)	2003 (%)	2003 (%)	2003–2004 (%)	2005 (%)	1998 (%)	2001 (%)	2004 (%)
Method :								
Number of prisons, inmates	135 prisons	134 prisons	3 prisons	23 prisons	9 prisons	All prisons		
	8 728 files	6 087 files	1 463 files	998 inmates	215 questionnaires	Questionnaires to medical staff		
Population	Incoming inmates,	Incoming inmates,	Incoming inmates	Cross sectional study Stratified random sample	Drug users' sample self-questionnaire	All inmates		
Study period	1 month	from 2 weeks to 3 months depending of the size of the prison	1 year			1 week		
diagnosis	Regular, extended drug use previous 12 months	Regular, extended drug use previous 12 months	Regular use, abuse or dependence during previous 6 months	DSM-IV criteria for drug abuse or dependence, including cannabis				
1. Heroin, morphine, opium use	14.4	6.5			15			
2. Cocaine/crack use	8.9	7.7	4.1 [1+2+3]	27.9 [1+2+3+ cannabis use]	26			
3. Other drugs (LSD, ecstasy)	3.4	4.0						
4. Psychotropic drugs use	9.1	5.4	2.5		67			
5. Polydrug use	14.6	10.5	29		65			
6. Intravenous drug use	6.2	2.6			10			
7. History of drug injection	11.8	6.5						
8. Methadone at prison entry	0.6	1.5			22			
9. Buprenorphine at prison entry	6.3	6.0			78			
10. OST at prison entry* or during prison stay**	6.9*	7.5*	11*		56*	2.0**	5.4**	6.6**

Lukasiewicz et al. [37] randomly selected 998 prisoners. Diagnoses were assessed using a structured interview (MINI 5 plus) [38]. They identified overall 35.2% of inmates as presenting either alcohol abuse and dependence (18.4%) or drug abuse and dependence (27.9%) with 11.2% (N = 111) presenting both conditions.

In the OPPIDUM project [39], a comparison was made in the 2005 study between subjects with a history of drug use in prison (215 subjects in 9 prisons) and primary care (248 subjects). Among the former, 65% had used more than one drug in the week preceding prison entry with a mean of 2.3 drugs (45% in primary care with a mean of 1.6 drugs). Ten percent reported drug injection in the week preceding prison entry (7% in the week before for those in primary care), 29% had sniffed (13% in primary care), 31% were alcohol dependant (6% in primary care), 48% had taken benzodiazepines (11% in primary care) and 4% had injected buprenorphine (7% in primary care).

#### Substitution treatments and psychotropic drugs at entry

In 2005, between 75,000 and 87,250 individuals in France were receiving buprenorphine and between 14,100 and 20,200 were receiving methadone. These 89,100 to 107,450 persons accounted for nearly 70% of the estimated opioid dependent population in France at that time [40].

At prison entry, 0.6% of inmates in 1997 and 1.5% in 2003 reported being on methadone treatment, while for buprenorphine these figures were 6.3% in 1997 and 6.0% in 2003 [33] (see table 2). While the proportion of inmates reporting persistent and regular use of opiates at admission decreased during this period (self-report, 14.4% in 1997 but 6.5% in 2003), access to substitution treatment appears to have improved (6.9% in 1997 vs. 7.5% in 2003). Inmates reported taking more anti-psychotics and anti-depressants at prison entry in 2003 than in 1997 (respectively 4.5% and 5.1% vs. 3.5% and 4.0%), but less anxiolytics or hypnotics (12.0% vs. 15.2% in 1997) [33]. In the Sahajian et al. study [36] conducted in 2003, 11% of the 1,463 prisoners at incarceration for whom information was available, had received OST before prison entry.

In the 2005 Oppidum study [39], 56% of drug users who answered the questionnaire had received OST (of which 78% were being treated with buprenorphine and 22% with methadone) in the week prior to prison entry. This figure was 85% in primary care (of which 71% were on buprenorphine and 29% on methadone).

### Drug injection in prison

Data regarding injection risk behavior in French prisons are limited. Rotily [41] carried out a survey in four prisons in the south and west of France. The survey was carried out in response to a request from the Ministry of Health and the Director of the correctional administration in 1997–1998. An anonymous questionnaire including questions on socio-demographic data, past sentences, drug use and sexual behaviors prior to and during incarceration, tattooing, access to medical care and past medical history was provided to all inmates. Overall, 72% of inmates agreed to participate by answering the questionnaire (1,212 subjects/1,695).

One hundred and fifty (13%) inmates reported having injected drugs at least once during their lifetime, of whom 103 (77%) reported being active injecting drug users (IDUs) during the previous 12 months. Forty five (30%) reported sharing needles or syringes during their last drug injecting episode.

Inside prison, 43 (42%) of the 103 inmates who were active IDUs before prison continued to inject in prison. Of these 21% (9) reported sharing needles or syringes during their most recent drug injecting episode. Seven inmates (7% of the 103 active IDUs before prison) reported having started injection practices in prison.

In a 2003 survey studying organization of OST provision in 22 French prisons [42], in more than half prisons (12/22), the prison staff (especially nurses) was aware of injection practices among prisoners.

In 2004 a national representative cross-sectional study of injecting drug users [43] found that 60% of the 1,462 drug users enrolled (i.e. those who reported sniffing or injecting at least once during lifetime) reported one or more experiences of incarceration. Among them, 12% reported injection drug use during their prison stay, of whom 30% reported having shared syringes or needles in prison.

### Other risk behavior reported by inmates

In the Rotily et al. study [41], 1% of the 1,212 male and female inmates who answered the questionnaire reported homosexual sex in prison, while 8% reported heterosexual sex. One percent reported accepting money for sex. Only 20% of the inmates who reported homosexual sex in prison reported condom use.

Almost a fifth (19%) reported being tattooed during their prison stay with significantly more IDUs (39%) reporting tattooing than non IDUs (18%).

### Suicide

Suicides dramatically increased in French prisons between 1990 and 2000 (12.3 suicides/10 000 inmates vs. 23.9/10 000 inmates)[44].

Data regarding suicide among inmates sentenced for drug offences are limited.

Of the 226 suicides among inmates in French prisons in 2001–2002, 15 involved inmates sentenced for drug offences (suicidal rate = 11.1/10 000 inmates). This is a lower rate than the mean inmate suicide rate (23.3/10 000 inmates) or the suicide rate for inmates incarcerated for criminal offences (77.2/10 000 for murderers, 46.1/10 000 for rapists)[45].

Data for overdoses inside prisons are not available but are also sparsely reported in the international literature.

### Post release follow-up

A study conducted in 2001 [46] evaluated the mortality rate of inmates in the first five years following release from a large prison in a suburb of Paris. Among 1,439 inmates released from January 1^st ^to December 31, 1997, information concerning mortality status was ascertained for only 1,245 inmates (86.5%). Seventy-one died between in 1997 and 2001, 35 of these (all men) during the first year after their release. Data from 14 of these inmates who had been transferred to this prison from other prisons for medical reasons (a penitentiary hospital being available) were excluded to avoid a selection bias. Twenty one inmates therefore who died during the first year after release (annual mortality rate = 1.8%) were included. Causes of death were known only for those who died in 1997 and 1998. Causes of death included overdoses (N = 4), alcoholic cirrhosis (N = 3), cardiovascular diseases (N = 3), suicides (N = 2), AIDS (N = 1), cancer (N = 1), respiratory disease (N = 1) and unknown causes (N = 6).

The Standard Mortality Ratio (SMR) for inmates to general population found a higher death rate for the released inmates (SMR = 321.3) [46] confirming the results reported in similar studies [47-50].

For inmates aged 15–34 years, the risk of drug overdose death was 120 fold greater than the general population while for inmates aged 35–54 years, the risk of drug overdose death was 270 times greater. Surprisingly, no drug overdose deaths were recorded during the first 2 weeks following prison release.

### Recidivism rate

According to the Ministry of Justice [51], in 2004, 33.8% (7 969) of the 23,550 subjects sentenced for drug related offences had been previously incarcerated, and 11.2% (2 645) had previously been incarcerated for drug related offences.

In the Regional Centre for Disease Control of South-Eastern France (ORS-PACA) study [41], 28% of the 150 IDU inmates reported at least 5 previous incarcerations and 49% had already spent more than 3 years in prison since 1980. Among the 978 non-IDU inmates, only 9% had previously experienced 5 or more incarcerations with 35% having spent more than 3 years in prison since 1980.

### Screening, prevention and health promotion

HIV prevention in prisons is regulated by a 1996 Ministry of Health/Ministry of Justice joint circular [52] and includes education, HIV and hepatitis screening, anti-retroviral post-exposure prophylaxis, access to HAART and hepatitis C treatments, bleach distribution, condom distribution, opioid substitution treatment (OST) and organization of follow-up after release. Unlike a number of other European countries, NSPs are still not permitted in French prisons.

According to the official harm reduction joint report from Ministry of Health and Ministry of Justice [53], the availability of education and staff training varies greatly from one prison to another.

By contrast, in the same report [53], HIV and hepatitis screening was considered to be satisfactory at prison entry and during detention although it was recommended to renew information and testing proposals more systematically and to improve the communication of results to inmates as there were still excessive delays between tests and results or inadequate communication of positive results.

No data could be found concerning HIV incidence among inmates or HIV outbreaks in French prisons.

### Access to HAART

HAART is available in all French prisons. Nevertheless, a national report from Ministry of health and Ministry of Justice [44] found that during 1994–2000, fewer HIV positive individuals were receiving anti-retroviral treatment in prison than in the general HIV-infected hospital population (73% vs. 88% in 2000), monotherapy was more common (20% vs. 12%) and multiple combination therapy less common (9% vs. 17%). However, these differences disappeared after adjustment for AIDS severity level (patients treated in reference HIV treatment centers having more advanced disease than prison inmates), suggesting comparable access to HAART inside and outside prison.

### Bleach distribution

Bleach is distributed to inmates every 2 weeks and can be purchased by inmates in prison inexpensively. According to the ORS-PACA study [54], only 59% of the active injecting drug inmates use bleach to disinfect their needles and syringes. The joint health-justice report on harm reduction [53] in French prisons concluded that the protocol needed to ensure the efficacy of bleach should be made more accessible to inmates although a recent report by WHO emphasized the lack of field evidence that bleach is effective in preventing HIV transmission among injecting drug users [55].

### Condom distribution

Condoms should be available in all medical units inside prisons and also be accessible in all other sites of the prison environment. Among the 25 prisons evaluated in the ORS-PACA study (1998)[54], condoms were only available in 23. In addition, 34% of inmates believed that condoms were not available in prisons, and 29% reported that they needed to ask doctors or nurses to obtain them.

### Substitution therapy

Methadone and buprenorphine have been widely used in France since 1996 as OST. In 2005, between 75,000 and 87,250 individuals in France were receiving buprenorphine and between 14,100 and 20,200 methadone [40]. Since 1996, both agents have been made available in French prisons for patients whose treatment was previously initiated outside prison. Until 2002, only buprenorphine could be initiated inside prisons except when authorized physicians (prescribing doctors in methadone programs) had been consulted by the patient. Since 2002, all hospital doctors (including doctors working in prison) have been authorized to initiate methadone in prisons.

The national report from the Ministry of Health and the Ministry of Justice [44], concluded that OST coverage in French prisons had been only increasing slowly in recent years because many doctors were not only reluctant to initiate OST in prison but were also to simply renew existing buprenorphine or methadone prescription. The proportion of inmates receiving OST increased from 2% in 1998 to 3.3% in 1999, 5.4% in 2001 and 6.6% in 2004 [56]. These proportions are comparable to those observed outside prisons if we take into account the estimated prevalence of drug use among inmates at prison entry 23% to 43% [57]. A study carried out in 2002–2003 [42] documented OST coverage in 22 prisons (accounting for 11,168 inmates, 20% of all French inmates at the time of the study). Most of the inmates were on remand and overall 7.8% (N = 870) were receiving OST, 81.5% with high dosage buprenorphine (N = 709) and 18.5% (N = 161) with methadone. Important variations in access to OST were observed between prisons with inmates on OST in small prisons accounting for only 2% of the total compared to 16% from larger prisons. Care provision and management including access to HAART and OST varied considerably between French prisons. Medical and prison staff expressed a preference for methadone, as daily delivery was easier to control and consequently resulted in less trafficking. Buprenorphine diversion (by injection or sniffing) and consequent trafficking was a major concern for prisons and medical staff. Inmates reported inadequate confidentiality and major stigmatization associated with daily delivery of OST.

In 2006, a national survey [58] was carried out to evaluate the impact of access to methadone initialization in all hospital outpatient services including prison medical services. The percentage of methadone patients among inmates receiving OST increased to 35% in 2006 from 22% in 2004; among patients receiving methadone, 60% initiated methadone treatment inside prison in 2006 (89.7% for buprenorphine initiation). Of the 98 prisons in total answering the questionnaire, physicians refused to initiate methadone prescription for "ethical" reasons in 3 prisons and for practical or organizational reasons in 8 others. In addition, in 12 prisons, the absence of methadone initiation was justified by the absence of indication. Among the total number of prescriptions of methadone inside prisons, 28% concerned initiation of methadone prescription.

## Discussion

These data indicate that the proportion of individuals incarcerated in France for drug-related offences is relatively high. Two thirds of sentences imposed for drug-related offences involve individuals arrested for illicit use of drugs or possession or acquisition of illicit drugs.

Although the duration of incarceration for these drug-dependent individuals may be relatively short, it seems likely that any delay in initiating OST for these individuals increases the chance of high risk injecting practices in prison. This was confirmed in two studies, one carried out in 2000 in prison [41] and the other in 2004 in the general community [43].

The first study clearly showed that approximately half (42%) of those reporting active drug use prior to incarceration continued to inject in prison, of whom 21% reported sharing needles or syringes in prison [41]. This result is consistent with findings in a more recent national representative survey enrolling drug users at different entry points (NSP, methadone buses, centers for drug users etc) which showed that, among those who practiced injection in prison, one third reported having shared syringes and needles in prison [43].

There are great difficulties in estimating the prevalence of injecting practices in prisons. This is partly due to the lack of recent data but also because of under-reporting. However, it seems that although injecting practices are less prevalent among prison inmates in France than among their counterparts in other countries [59], a considerable portion of inmates are still at high risk of blood-borne viral infections.

In addition, considering that the prevalence of HIV in these populations is around 11%, HIV-infected individuals at prison entry do experience at least 2 day interruptions of their HAART, especially if prison entry occurs during the week-end.

It is widely known that these interruptions considerably increase the risk of developing resistance [60,61] with a consequently high probability of circulation of HIV resistant strains in prison settings.

The continuity of HIV care for inmates remains a major problem which is strongly related to the risk of stigmatization in prison and the problem of social integration after release.

It has major public health implications, and is becoming reported more frequently [62,63], especially since the introduction of HAART regimens which are "less forgiving" (i.e. requiring higher adherence to obtain viro-immunological response) and which can increase the risk of virological failure [63] or resistance in re-incarcerated individuals due to reduced adherence or treatment interruptions after release. HIV care needs to commence within the first day of incarceration and post-incarceration care needs to be arranged before release.

Compared with other European countries [59] in a cross-sectional European survey, HIV prevalence in French prisons (2.2%) was situated just between that found in south-European countries (6.2% in Italia, 16.7% in Portugal, 12.9% in Spain) and in north-European countries (0.7% in Germany, 1.6% in Sweden, 1% in Scotland, 0% in Belgium). The same result existed for HCV (8.3%) between south-European countries (24% in Italy, 34.1% in Portugal, 46.7% in Spain) and north-European countries (4.9% in Germany, 10.9% in Belgium).

The situation concerning OST access is slowly improving but there is still an important heterogeneity of care between prisons and insufficient coverage of inmate needs [42]. According to the European Network of Drug Services in Prison (ENDSP) report in 2004 [64], an important heterogeneity also exists between European countries and inside many European countries themselves. A treatment gap persists between those requiring substitution treatment and those receiving it and, in most of the countries studied, coverage is irregular. In 2004 Greece and Sweden still did not offer treatment in prisons. In most countries, treatments are discontinued or dosages reduced when someone enters prison. In some countries, OST are limited to a period of between 6 to 12 months.

Its role in facilitating delivery of antiretroviral therapy to IDUs should be given greater recognition in prisons [65].

Despite the availability of OST in French prisons, the lack of access to NSP means that inmates who are still injecting while incarcerated are at high risk of HCV or HIV seroconversion. The introduction of NSP in prison is urgent and is also justified by recent data [66] showing that access to both methadone and NSP has an impact on HCV seroconversion. However, despite WHO support for the strong evidence base for prison NSPs [55], little headway has been achieved in France in the debate about their introduction. This may be due to the following reasons: firstly, as some inmates are incarcerated only because of their illegal drug consumption, allowing access to NSPs inside prison would highlight the limited effectiveness of incarceration in the promotion of abstinence and would also draw attention to the inadequacy of a drug policy heavily reliant on supply control. This could prompt many community members to consider alternatives to a policy dominated by drug law enforcement. Secondly, NSPs are still regarded by the correctional staff and authorities as "weapons in inmates' hands".

This is quite surprising if we consider that access to NSPs is readily available in community settings in France and that such access has greatly contributed to the reduction of HIV prevalence among IDUs [28]. NSPs are already available in Switzerland, Germany, Spain, Luxembourg and Scotland, and will soon become available in Portugal and in a growing number of developing countries [55].

Data about HIV, HCV and HBV prevalence at prison entry are difficult to interpret because they are either based on self-report or on testing of those who agreed to be tested (and therefore may bias estimates of prevalence). The higher proportion of individuals tested for HCV is attributable to more active testing, due to the availability of HCV treatment in prisons. Interestingly, a three-fold increase in the proportion of individuals vaccinated against HBV was observed between 1997 and 2003, but it is not yet known to what extent this reflects changes in the general population of people at risk of HBV seroconversion or is due to a change in the characteristics of individuals entering prison.

Assessment of alcohol dependence at prison entry is insufficiently emphasized at present as one third of inmates report excessive alcohol consumption, only sometimes associated with drug dependence. However the proportion of individuals who are recent IDUs at prison entry seems to have decreased over the past years, probably reflecting wider access to OST but also a change from injecting to less harmful routes of administration (such as sniffing or snorting) in the community. Mortality after release, mostly due to drug overdoses, is high and comparable in France to results found in similar studies for other countries [67]. The post prison release period is usually considered a very risky time for overdose as already shown in other studies [68].

The increasing use of psychotropic drugs among prison entrants suggests the importance of providing comprehensive care in prison settings with psychiatrists and psychologists possibly playing a major role in the identification and management of psychiatric co-morbidities and alcohol and drug dependence but also in HIV or HCV treatment related side effects.

The existence of unsafe sexual behaviors during incarceration and undervalued importance of the high prevalence of tattooing suggests the need for additional preventive measures [69].

The high recidivism rate of IDUs and consequent rapid cycling in and out of prison almost certainly contribute greatly to the transmission of blood-borne infections (including viral resistant strains) from prison to the general population.

Moreover, access to care is still inadequate and services increasingly stretched by an ever growing prison population and the high prevalence of co-existing severe mental and other health and social problems which exacerbate the difficulties in providing a comprehensive health approach in prison settings [37,70]

Some recommendations can be outlined from these data. Access to OST in prison requires improvement in monitoring standardized approaches to ensure equity of access in prison. Similarly, condom distribution should be expanded to all areas of prisons to ensure confidentiality and avoid stigma. In addition, access to post-exposure prophylaxis in the event of sexual or parenteral exposure should be promoted to ensure access is comparable to that for the general population. Health authorities need to become more sensitive to the problems of HAART interruption as these may not only induce failures of HIV treatment in inmates but may also contribute to the circulation of HIV resistant strains both inside and outside prisons.

Effective, evidence-based preventive measures in prison settings may reduce harm resulting from multiple incarcerations or long periods of imprisonment.

## Conclusion

The large gap in France between health prevention and treatment services in the community and the equivalent services for prison inmates cannot be defended.

This set of indicators, though limited and often outdated, clearly highlights the need for more research in this field in order to both obtain accurate estimates of HIV-HCV occurrence and risk behaviors in French prisons, and carry out interventional studies to identify which models can assure continuity of care and appropriate social services after release.

Irrational hostility to prison NSPs must be overcome by authorities so that pilot studies can be commenced in a few prisons to demonstrate their feasibility in the French prison system.

Introducing preventive and harm reduction measures not yet available in French prisons is not only a human right for prison inmates but can also provide important public health benefits for the general population.

## Declaration of competing interests

The authors declare that they have no competing interests.

## Authors' contributions

LM collected the data and wrote the results, MPC and LM wrote the introduction and the discussion and revised the entire manuscript, AW participated in the design of the review, contributed to the discussion and revised the entire manuscript.

All authors read and approved the final manuscript.
